# Short-Term Impacts of Diets Containing an *Aloe vera* Peel Water Extract on Hypoxia Tolerance and Biochemical Markers in *Cyprinus carpio* var. Jian

**DOI:** 10.3390/ani16152421

**Published:** 2026-08-05

**Authors:** Jia Yuan, Dan Gou, Qiqi Li, Jianglong Liao, Jing Xu, Qihui Yang, Gangfu Chen, Huatao Li

**Affiliations:** 1 College of Fisheries, Neijiang Normal University, Neijiang 641100, China; yuanjia0621@163.com (J.Y.); gd1951875973@163.com (D.G.); lqq7157152026@163.com (Q.L.); 18708437541@163.com (J.L.); 10001934@njtc.edu.cn (J.X.); fanyahr@163.com (G.C.); 2 Fishes Conservation and Utilization in the Upper Reaches of the Yangtze River Key Laboratory of Sichuan Province, Neijiang Normal University, Neijiang 641100, China; 3 College of Fisheries, Guangdong Ocean University, Zhanjiang 524088, China; qihuiyang03@163.com

**Keywords:** *Aloe vera* peel, hypoxia tolerance, biochemical indicators, carp, antioxidant

## Abstract

This study aimed to assess how dietary water extract of *Aloe vera* peel (WEA) affected biochemical markers and hypoxia tolerance in Jian carp (*Cyprinus carpio* var. Jian). 630 Jian carp (11.77 ± 0.10 g) were fed diets with increasing WEA concentrations (0.0–1.8%) for 15 days. The results showed that short-term feeding of WEA enhanced carp growth performance (*p* < 0.05). When WEA was added to diets in hypoxic conditions, carp’s duration time (DT) increased and their oxygen consumption rate (OCR) decreased (*p* < 0.05). In carp blood, feeding WEA improved hematological parameters, such as hematocrit, hemoglobin concentration, mean corpuscular volume, and mean corpuscular hemoglobin content, (*p* < 0.05) and decreased plasma ammonia levels. Lactic dehydrogenase levels in muscles and gills and transaminases (GOT and GPT) in red blood cells, gills, and hepatopancreas were significantly higher in carp fed diets containing WEA (*p* < 0.05). Additionally, dietary WEA decreased ROS generation and lipid oxidation in carp respiratory-related tissues and cells by raising both enzymatic antioxidant activities and non-enzymatic antioxidant levels (*p* < 0.05). Finally, dietary WEA increases hypoxia tolerance in carp by modifying protein and carbohydrate metabolism and boosting the antioxidant capacity of respiratory-related tissues and cells, suggesting that WEA may be a natural method of lowering hypoxic stress in fish.

## Simple Summary

Since adequate oxygen levels primarily enable fish aerobic metabolism, dissolved oxygen (DO) is a critical limiting factor in fish farming. Fish experiencing hypoxia and the ensuing reoxygenation processes are severely limited by the seasonal and daily fluctuations in oxygen content in freshwater habitats. Because of its numerous health benefits, *Aloe vera* has long been used for apparent growth-promoting, antimicrobial, antioxidative, and immunomodulatory properties. Thus, this study examines how water extract of *Aloe vera* peel (WEA) affects carp (*Cyprinus carpio* var. Jian) hypoxia tolerance and biochemical markers. Our research indicates that short-term dietary WEA can naturally promote fish growth and prevent fish stress brought on by hypoxia, offering a possible dietary approach to reducing fish resilience in low-oxygen environments.

## 1. Introduction

According to the description of Abdel-Tawwab et al. (2019) [1], dissolved oxygen (DO) is essential for supplying oxygen to tissues, promoting aerobic metabolism in the fish body, and having a major impact on fish farming. Fish kept in captivity often encounter recurrent and persistent stressors, such as periods of hypoxia followed by reoxygenation [2,3]. Hypoxic conditions in aquaculture may have detrimental effects on fish physiology and metabolism [4,5]. Studies have shown that hypoxia-reoxygenation not only impairs growth performance but also inhibits fish antioxidant defense mechanisms [6,7,8]. Investigating strategies to mitigate the detrimental impacts of hypoxic stress is therefore essential.

Natural products, such as polysaccharides, saponins, flavonoids, and active peptides, have been shown by Liu et al. to be beneficial in the anti-hypoxic treatment of high-altitude disease [9]. The antioxidant qualities of herbs are closely linked to their capacity to counteract hypoxia [9]. Juvenile tambaqui (*Colossoma macropomum*) are less affected by hypoxic assault when fed diets rich in ascorbic acid, a common antioxidant [10]. *Aloe vera* is known for its numerous health-promoting properties, such as immunomodulatory, pro-healing, antioxidant, antimicrobial, anti-inflammatory, and anti-toxic effects [11,12]. *Aloe vera* is rich in anthraquinones (aloin and aloe emodin), polysaccharides, saponins, amino acids, vitamins, etc. [13]. *Aloe vera* leaves can be separated into gel and peel. The food, pharmaceutical, and cosmetics industries now heavily rely on the aloe gel industry [13]. Despite making up more than half of the leaf’s weight, the peel is mostly thrown away as a by-product of processing *Aloe vera* [13]. *Aloe vera* polysaccharides, which are water-soluble components, have been shown to have antioxidant effects in rats [14] and to reduce oxidative damage in animals with oral ulcers [15]. Thus, the hypothesis of this study is that fish hypoxic stress may be reduced by the water extract of *Aloe vera* peel (WEA). However, nothing is known about how WEA affects fish hypoxia tolerance.

Carp is one of the most commonly farmed fish species in China, and it is renowned for its rapid growth, strong resistance to disease, and high degree of environmental adaptation [16]. Jian carp has become more well-liked by customers in particular due to its excellent flavor, low cost, and high nutritional content [17]. In order to provide vital information for its application as a natural cure to enhance hypoxia resilience in aquaculture systems, the purpose of this study is to examine the potential protective impact of WEA in reducing hypoxia stress in Jian carp.

## 2. Materials and Methods

### 2.1. Preparation of WEA

*Aloe vera* peels used in the study were purchased from a Chengdu First Pharmaceutical (Chengdu, Sichuan, China). After being dried, the peels were ground into a fine powder with particles smaller than 0.40 mm. The method outlined in the literature [3] was used to extract 500 g of these powdered peels. 4500 mL of petroleum ether, ethyl acetate, ethanol (analytical reagent (AR) grade, Chengdu Kelong Chemical Reagent Factory, Chengdu, China), and water were used sequentially in the extraction process. Each solvent was extracted for six hours at 800 rpm using an agitator (Model OS40-S, Dalong, Beijing, China). After the extraction, the solutions were initially evaporated at low pressure using a rotary evaporator (Model RE-52CS, Jinye, Shanghai, China). A concentrated solution of WEA was created by evaporating the water solution. After that, the concentrated solution was placed in a blast drying oven (Jinghong DHG-9030A, Shanghai, China) and kept at 50 °C without stirring until a stable dry product was obtained. The dried extracts were kept in sealed, light-shielded containers at −20 °C until further analysis.

### 2.2. Composition Analysis of WEA

At a temperature of 105 °C, dry matter (moisture) was measured using weight loss drying [18]. According to the study of Li et al. (2020) [19], the phenol-sulphuric acid method and the 3,5-dinitrosalicylic acid (DNS) method were used to determine the amounts of total sugar and reducing sugar in WEA using glucose as the standard. Polysaccharide content was calculated by subtracting reducing sugar from total sugar. The chemical components of WEA, such as polysaccharides, total sugar, reducing sugar, and dry matter, were summarized in Table 1.

### 2.3. Animal Experiment Diets

A thorough method for preparing the experimental diets was created by our study team [20]. All solid feed ingredients were coarsely crushed and sieved through a 60-mesh screen prior to formulation. The dry feed ingredients were then gradually combined in accordance with the suggested ratios. The oils (fish oil and corn oil) and WEA were then evenly mixed with the dry powder ingredients and sieved through a 40-mesh sieve to ensure even integration. After that, the mixture was thoroughly combined with water in an amount equal to 45% of the dry matter weight. A feed pelletizer was used to pelletize the feeds, which were then placed on drying racks and roasted at 50 °C. Before being used again, the dried feed pellets were placed in plastic bags, allowed to cool to ambient temperature, and kept at −20 °C.

The diet consisted of 5.33% crude fat and 34.89% crude protein. At concentrations of 0.0 (control), 0.3, 0.6, 0.9, 1.2, 1.5, and 1.8%, WEA was added to the baseline diet. Less wheat flour was utilized to make up for the WEA contribution. When doing proximate diet assessments, the AOAC’s guidelines were followed. The composition of the experimental diets is displayed in Table 2.

### 2.4. Feeding Trial

The juvenile *Jian carp* was bred on a fish farm in Neijiang, China. In order to accurately resemble their natural habitat, their surviving environment was meticulously recreated in the laboratory [3]. According to the description of Ceylan et al. (2012) [21], a temperature of 21.5 ± 1.5 °C was maintained in the water as a crucial environmental element influencing fish growth and feed efficiency. Every day, dechlorinated water was provided, and a natural light cycle was preserved utilizing tap water as the water supply while adhering to pertinent water quality standards. The following parameters were maintained: DO > 6 mg L^−1^, nitrite < 0.05 mg L^−1^, ammonium-nitrogen < 0.4 mg L^−1^, and pH 7.8 ± 0.3 units. An Octaderm W-II water quality analyzer was used to measure the water quality every two days, and a mercury thermometer was used to measure the temperature. A baseline diet was given to the fish. The tanks were randomly assigned to seven experimental groups of 630 juvenile *Jian carp*, with an average weight of 11.77 ± 0.10 g. Three aquariums, each with thirty fish, were given to each group. While the control group was fed a basic diet, six of the seven treatment groups received diets supplemented with 0.3, 0.6, 0.9, 1.2, 1.5, and 1.8% percent WEA. Throughout the 15-day trial, all fish groups were fed four times a day, and a careful examination was done to make sure they were satisfied without being overfed.

After a 24 h fast, fish in each aquarium were gradually numbered and weighed at the end of the rearing experiment. The beginning number, final number, initial body weight (IBW), final body weight (FBW), and feed consumed were used to calculate the weight gain rate (WGR) [22], feed efficiency (FE), feed intake (FI) [23], and protein efficiency ratio (PER) [18].

WGR (%) = [FBW (g fish^−1^) − IBW (g fish^−1^)]/IBW × 100

FI (g fish^−1^) = [feed offered (g) − feed residues (g)]/final number of fish

FE (%) = 100 × WG/FI

PER = weight gain/(FI × dietary protein content)

### 2.5. Hypoxia Trial

At the end of the feeding experiment, fish (10 per tank) were weighed for body weight (BW, g) in accordance with our earlier study [3]. In the aquarium, 60 L of fresh tap water was continuously oxygenated for 24 h using an aerator (SB-748, Shanghai Sunshine Pump, Shanghai, China) to reach room temperature (22 °C) and a saturated DO condition. A Leici JPBJ-608 dissolved oxygen analyzer (INESA Scientific Instrument Co., Ltd., Shanghai, China) was used to measure the initial DO (IDO, mg L^−1^) in the saturated DO water. The water volume (WV, L) of 33 times the fish’s BW was carefully transferred to a necked container (polyethylene terephthalate, 4.5 L capacity) prior to the measurement. The bottle was filled with ten fish of known weight. By applying pressure to the bottle wall, the extra air was expelled. The timer was set and the bottle mouth was shut right away. The timer was stopped when eight fish in the experimental bottle lost equilibrium, and the water’s final DO (FDO, mg L^−1^) was determined right away. The duration of hypoxia (DT, h) was defined as the time interval between the bottle mouth closing and the fish losing its equilibrium. The amount of DO in the bottle’s water dropped from 8.14 mg L^−1^ to 0.10 mg L^−1^ during this operation. The following formula was used to get the fish body’s oxygen consumption rate (OCR, mg g^−1^ h^−1^).

OCR = (IDO − FDO) × WV/(DT × BW)

### 2.6. Sampling

The sample collection procedure was conducted according to the guidelines established by our research group [24]. Ten fish were put to death by being placed on ice after being anesthetized with a 50 mg L^−1^ benzocaine solution in water. Fish blood samples were taken from each group by caudal puncture and placed into heparinized syringes. The blood’s hematocrit (Hct), red blood cell count (RBC), and hemoglobin (Hb) concentration (HbC) were determined using techniques outlined in the literature [25]. The mean corpuscular hemoglobin concentration (MCHC) (HbC divided by Hct), mean corpuscular hemoglobin content (MCH) (HbC divided by RBC), and mean corpuscular volume (MCV) (Hct divided by RBC) were computed using these measures [25].

Within an hour, the blood was centrifuged at 1000× *g* for three minutes at 4 °C. Red blood cells and plasma were isolated from the blood. Plasma was collected in order to determine the ammonia content (PAC). The red blood cells were kept at −80 °C to measure the amounts of superoxide anion (O_2_^·−^), reduced glutathione (GSH), hydrogen peroxide (H_2_O_2_), glutamate-pyruvate transaminase (GPT), and glutamate-oxaloacetate transaminase (GOT). All internal organs were then removed from the abdominal cavity. GOT, glutathione peroxidase (GPx), and GPT activities as well as H_2_O_2_ concentration were determined in hepatopancreas tissues after the intestines were swiftly removed from the organs, snap-frozen in liquid nitrogen, and kept at −80 °C. Intestinal tissues were examined for hydroxyl radical (·OH) levels, as well as catalase (CAT) and superoxide dismutase (SOD) activity. In a similar manner, the gills were promptly removed and kept at −80 °C for tests that evaluated the levels of H_2_O_2_ and malondialdehyde (MDA), as well as the activities of GOT, CAT, GPT, and lactate dehydrogenase (LDH). The muscle was quickly removed and stored at −80 °C in order to perform the assays for glutathione reductase (GR), CAT, GOT, and LDH activities as well as MDA level. The Nanjing Jiancheng Bioengineering Institute (Nanjing, China) provided the reagent kits utilized in the experiment.

### 2.7. Ethics Statement

The procedures described above were conducted in accordance with the ethical guidelines set by the Institutional Ethics Committee of the Chinese Institute of Chemical Biology. The Institutional Animal Care and Use Committee at Neijiang Normal University granted approval for the experiments to be conducted, ensuring that the study met relevant ethical standards and laws pertaining to the humane treatment of animals.

### 2.8. Biochemical Analysis

The tissue samples were homogenized using nine parts (*w*/*v*) of cold physiological saline. This homogenate was then centrifuged for 20 min at 4 °C and 3200× *g*. The resulting supernatant was kept in order to examine various enzymes, such as GOT and GPT activity [26]. Furthermore, measurements of SOD, CAT, GPx, and LDH activities as well as O_2_^·−^, ·OH, and GSH levels were examined using known techniques [3,27]. The H_2_O_2_ level was measured using the procedure outlined in the literature [28]. The methodology used in the literature [29] was to measure the MDA level. The paper [25] states that GR activity was computed. Total soluble proteins were evaluated using the Bradford method [30]. The publication [31] states that Hb content was measured.

### 2.9. Statistical Analysis

All experimental results were expressed as mean ± standard deviation (S.D.) to show the data’s central tendency and dispersion. Software called SPSS 22.0 was used for statistical analysis. One-way ANOVA was used to evaluate significant differences between the experimental groups after these presumptions were met. Duncan’s multiple range test was used for post hoc comparisons to identify certain group pairs with significant differences if the ANOVA results indicated significant differences (*p* < 0.05).

## 3. Results

### 3.1. Impact of Dietary WEA on the Jian Carp’s Growth Performance

**Figure 1:**
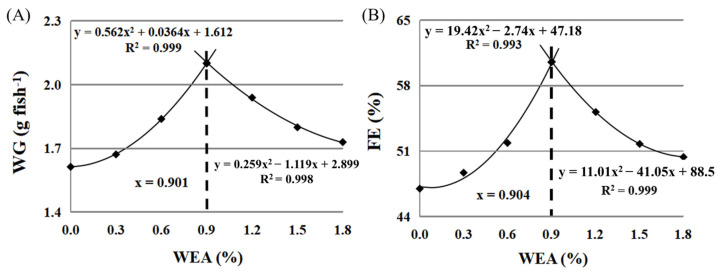
Weight gain (WG) (**A**) and feed efficiency (FE) (**B**) of *Jian carp* fed varying amounts of water extract of *Aloe vera* peel (WEA) for 15 days were analyzed using broken-line regression. The values are the averages ± S.D. of three experiments, each containing thirty fish (*p* < 0.05).

The results of supplementing *Jian carp* with different quantities of WEA on their growth performance are shown in Table 3. When compared to those who did not receive the medication, WEA supplementation significantly changed growth performance by improving the FBW, WGR, FE, and PER (*p* < 0.05; Table 3). No deaths occurred among the experimental subjects. WGR, FE, and PER were the highest in fish fed a diet containing 0.9% WEA; however a decline was observed following 1.2% WEA supplementation (Table 3). The ideal dietary intakes of WEA for weight gain and FE were 0.901% and 0.904%, respectively, according to broken-line regression analysis (Figure 1).

### 3.2. Impact of Dietary WEA on Jian Carp’s Resistance to Hypoxia

**Figure 2:**
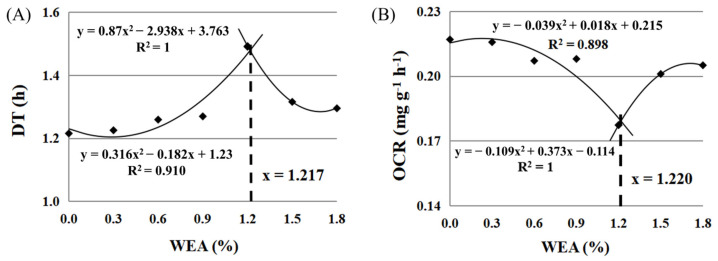
Broken-line regression was used to examine the durative time (DT) (**A**) and oxygen consumption rate (OCR) (**B**) of carp given different concentrations of Aloe vera peel water extract (WEA) for 15 days. The values are the mean ± S.D. of three replicates with 10 fish in each (*p* < 0.05).

Carp fed the diets supplemented with WEA had a substantially longer hypoxic DT (*p* < 0.05; Table 4) and a significantly lower OCR (*p* < 0.05; Table 4) than the control group. Table 4 shows that carp fed a diet containing 1.2% WEA under hypoxia had the greatest DT and lowest OCR (*p* < 0.05). The best additions for hypoxia tolerance were 1.217% and 1.220% WEA, respectively, according to the regression analysis of DT and OCR (Figure 2).

### 3.3. Impact of Dietary WEA on Biochemical Index in Intestines and Hepatopancreas of Jian Carp

In contrast to the control, SOD and CAT activity in the intestines of WEA-treated carp first increased and then decreased, peaking at 1.2% and 0.6% WEA, respectively (*p* < 0.05; Table 5). The amount of ·OH in the intestines of carp treated with WEA first reduced and then increased (*p* < 0.05), with 0.9% WEA exhibiting the lowest values (Table 5).

**Figure 3:**
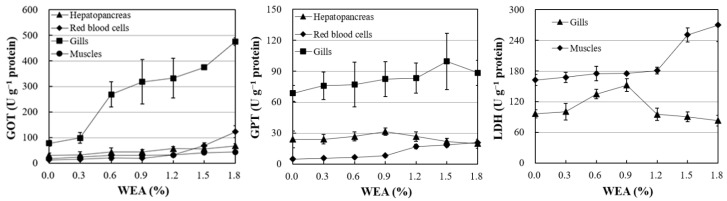
Effects of dietary water extract from *Aloe vera* peel (WEA) on the metabolic indicators in the hepatopancreas, red blood cells, gills, or muscles of *Jian carp*. The values represent the means ± S.D. of three replicates. GOT, glutamate-oxaloacetate transaminase; GPT, glutamate-pyruvate transaminase; LDH, lactate dehydrogenase.

When compared to the control, GOT activity in the hepatopancreas of *Jian carp* was significantly raised by feed of WEA supplementation, and it increased gradually as dietary WEA concentrations increased (*p* < 0.05; Figure 3). The activities of GPT and GPx in the hepatopancreas of carp treated with WEA showed a tendency to first increase and then decrease (*p* < 0.05), peaking at 0.9% and 1.5% WEA, respectively (Table 5; Figure 3). When compared to the control, the H_2_O_2_ level in the hepatopancreas of *Jian carp* fed WEA supplementation was significantly lower, and it decreased continuously as dietary WEA concentrations decreased (*p* < 0.05; Table 5).

### 3.4. Dietary WEA’s Effects on the Hematological and Hematochemical Indicators of Jian Carp

Following the addition of 0.9% WEA, Table 6 demonstrated that the Hct, HbC, MCV, and MCH were significantly higher than in the control group, reaching their maximum value (*p* < 0.05). The aforementioned parameters dropped (*p* < 0.05; Table 6) after the supplemental amount of WEA increased. RBC and MCHC were not considerably affected by varying dietary WEA supplementation dosages (Table 6). PAC first fell and then rose in response to rising dietary WEA levels, peaking at 1.2% WEA addition in *Jian carp* (*p* < 0.05; Table 6).

### 3.5. Dietary WEA’s Effects on the Biochemical Indicators in Red Blood Cells, Gills, and Muscles of Jian Carp

The GOT and GPT activities gradually increased as the amount of WEA given to *Jian carp* red blood cells increased (*p* < 0.05; Figure 3). The O_2_^·−^ and H_2_O_2_ concentrations were higher than in the control group at 0.9% and 0.3% WEA addition (*p* < 0.05; Table 7) and reached their lowest values at 1.2% and 0.9% WEA addition, respectively. The GSH level was higher than that of the control group at 0.9% WEA addition (*p* < 0.05), peaked at 1.5% WEA addition (Table 7), and then decreased as the amount of additional WEA rose (*p* < 0.05; Table 7).

Dietary WEA supplementation significantly increased GOT activity at 0.6% WEA addition compared to the control, and this activity gradually increased as WEA concentrations in diets increased in *Jian carp* gills (*p* < 0.05; Figure 3). LDH activity was considerably higher than in the control group at 0.6% WEA addition (*p* < 0.05), peaked at 0.9% WEA addition (*p* < 0.05), and then decreased at increasing WEA levels (*p* < 0.05; Figure 3). GPT and CAT activities were significantly higher than in the control group. They declined as WEA addition increased after peaking at 1.5% and 0.6% WEA addition, respectively (*p* < 0.05; Table 7; Figure 3). When compared to the control group, the H_2_O_2_ and MDA contents were significantly lower at 0.6% and 0.3% WEA addition, respectively (*p* < 0.05), reached their lowest value at 0.9% WEA addition, and then increased as the WEA addition increased (Table 7).

Dietary WEA supplementation greatly increased GOT and LDH activities in comparison to the control at 0.6% and 1.5% WEA addition, respectively (*p* < 0.05; Figure 3). These activities progressively increased when WEA concentrations in the diets increased in the *Jian carp* muscles (Figure 3). CAT and GR activities first increased in *Jian carp* muscles before decreasing (Table 7). CAT activity was significantly higher than that of the control group (*p* < 0.05) and peaked at 1.2% WEA addition. When compared to the control group, GR activity rose considerably (*p* < 0.05) at 0.3% WEA addition and peaked at 0.9% WEA addition. The MDA level in *Jian carp* muscle showed a tendency of first decreasing and then increasing (Table 7). The level of H_2_O_2_ was substantially lower than in the control group at 0.9% WEA addition (*p* < 0.05) and when the diet’s 1.2% WEA addition reached the minimum (Table 7).

## 4. Discussion

### 4.1. Fish Growth Performance Was Enhanced by Dietary WEA

FE, WGR, and PER can be used to represent fish growth performance [32,33]. The current findings were consistent with earlier research showing that an 80% methanol extract of *Aloe vera* enhanced *Oreochromis niloticus* weight gain and feed conversion ratio [34]. According to the current research, showing that WEA improves fish growth performance. Broken-line regression analysis shows that fish had the maximum levels of FE and weight increase at 0.904% and 0.901% inclusion levels of WEA in the diet, respectively.

Fish growth is positively correlated with digestion and absorption capacity [35]. For fish to absorb and digest nutrients, the intestines and hepatopancreas are vital digestive organs [36]. Reactive oxygen species (ROS) such as O_2_^·−^, H_2_O_2_, and ·OH oxidize biological components such as proteins and lipids, including essential enzymes [37]. Polysaccharides accounted for up to 28.84% of WEA. The present study in the intestines and hepatopancreas of carp was consistent with the previous discovery that *Aloe vera* polysaccharides considerably reduced O_2_^·−^ and H_2_O_2_ in vitro [14]. The hypothesis that eating WEA inhibits the formation of ROS in fish digestive organs is supported by these results. The effects of WEA on ROS may be intimately associated with fish antioxidant systems. Fish have evolved enzymatic antioxidants to fight ROS in their tissues and organs [38]. SOD, a crucial part of antioxidant systems, transforms O_2_^·−^ into H_2_O_2_ and O_2_ [39]. H_2_O_2_ is then detoxified by enzymes including GPx and CAT [40]. The SOD, CAT, and GPx activities in the carp’s intestines or hepatopancreas in this investigation were in line with the discovery that giving rats *Aloe vera* polysaccharides greatly raised cardiac SOD activity [14]. These results show that dietary WEA can scavenge ROS by increasing enzymatic antioxidant activity in fish digestive organs.

### 4.2. Fish’s Ability to Withstand Hypoxia Was Improved by Dietary WEA Supplementation

According to reports, fish growth performance has declined as a result of hypoxia [6,41,42]. DO is a major limiting factor in fish farming because enough oxygen levels are required to maintain fish aerobic metabolism [1]. Fish subjected to hypoxia and subsequent reoxygenation events in freshwater settings have difficulties due to significant seasonal and daily changes in oxygen concentrations [43]. According to the current study, dietary WEA supplementation improved DT in hypoxic carp, increasing their tolerance to hypoxia. The effects of WEA on fish in hypoxic conditions are not well studied. Fish typically alter their metabolic rate in low-oxygen environments [44]. OCR is a significant indicator of aerobic metabolic activity [45,46]. Metabolic rate depression is a significant physiological adaptation that enhances long-term survival in hypoxic environments [47]. The current study found that dietary WEA reduced OCR in hypoxic carp, indicating that dietary WEA may improve fish hypoxia tolerance by reducing aerobic metabolic activity. Using broken-line regression analysis of DT and OCR, the optimal WEA addition levels for carp were determined to be 1.217% and 1.220%, respectively.

Fish use aerobic protein metabolism as their main energy source [23,48]. GOT and GPT are key enzymes in fish protein metabolism [49,50]. Ammonia is the main by-product of protein breakdown in teleosts [51]. The findings demonstrated that administering WEA to carp reduced PAC while increasing GPT and GOT activities in the muscle, gills, hepatopancreas, and red blood cells. There is currently no published study on how WEA affects the regulation of PAC in fish or the activity of these crucial enzymes in the major metabolizing organs. These new results offer crucial insights into how dietary WEA supplementation may lower fish amino acid catabolism.

Dietary carbohydrates are vital for providing energy for healthy growth in animals [52]. LDH, a crucial enzyme in anaerobic glycolysis, encourages the metabolism of glucose to provide energy [37]. LDH activity in the carp’s muscles and gills was increased in this study by dietary WEA supplementation. Previous research has not looked at how WEA affects LDH activity in fish metabolizing organs. The current research indicates that dietary WEA improves fish glucose metabolism and energy supply. Animals in hypoxic conditions are known to use anaerobic glycolysis as their main energy source [47,53]. Thus, WEA may enhance fish hypoxia tolerance by increasing anaerobic glycolysis and reducing protein catabolism and OCR. Further research is required to elucidate the mechanisms via which WEA enhances fish hypoxia resilience.

The function of fish red blood cells may be connected to the impact of WEA on their ability to withstand hypoxia. Among the essential functions of red blood cells are the transfer of oxygen and the elimination of CO_2_ during breathing [54]. In red blood cells, hemoglobin serves as a protein that carries oxygen; it binds to oxygen to form oxygenated hemoglobin, which subsequently releases oxygen into the animal tissues [3]. In this study, dietary WEA increased the levels of Hct and HbC in carp blood. Accordingly, an 80% methanol extract of *Aloe vera* raised HbC in *Oreochromis niloticus* blood [34]. These findings suggest that WEA increases O_2_ transport via increasing the hemoglobin content of fish blood. The effect of WEA on the hematological index may be correlated with the age of fish red blood cells. In this study, WEA supplementation led to an increase in MCH and MCV in carp red blood cells. According to research, fish with mature red blood cells had higher hemoglobin levels and larger volumes than fish with younger red blood cells [55]. The results confirm that WEA consumption improves O_2_ transport by promoting the growth of fish red blood cells. The aforementioned findings demonstrate how dietary WEA may enhance fish red blood cell function.

### 4.3. Dietary WEA Supplements Improved Antioxidant Capacities in Respiratory-Related Tissues and Cells of Fish

Hypoxia causes oxidative damage in fish, according to earlier observations [6,7,8]. In addition to their primary function of delivering oxygen, red blood cells may also contribute to oxidative equilibrium, which makes them vulnerable to damage from ROS. O_2_^·−^ can be produced by the spontaneous oxidation of oxygenated Hb [56]. O_2_^·−^ dismutation may contribute to the formation of H_2_O_2_, which then reacts with heme Fe^2+^ to form ·OH [54]. Reduced O_2_^·−^ and H_2_O_2_ levels in the red blood cells of carp fed WEA were found in the current study, indicating a decrease in ROS formation. Antioxidants are crucial for maintaining the health and performance of tissues and organs in aquatic organisms [57]. Fish have non-enzymatic antioxidants like GSH to combat intracellular ROS [38]. The current study in carp red blood cells was consistent with previous research showing that GSH levels in rat heart tissues were elevated by dietary polysaccharides from *Aloe vera* [14]. The effects of WEA on these variables in fish red blood cells have not been thoroughly studied. Our results suggest that dietary WEA supplementation can reduce ROS generation in fish red blood cells by increasing non-enzymatic antioxidant levels.

The fish gill, which serves as both an osmoregulatory and respiratory organ, is particularly vulnerable to ROS damage due to its continuous exposure to environmental stress and high concentration of hemoglobin and red blood cells [58]. ROS can inactivate vital enzymes and induce biological harm in fish cells [37]. Lipid oxidation, demonstrated by elevated MDA levels, is a key sign of ROS-induced damage [39]. MDA levels in *Mugil cephalus* gills have been found to increase in response to hypoxia [59]. Fish muscles, especially the red muscles, are rich in myoglobin, the primary tissue employed for O_2_ storage and utilization [60]. Previous research indicates that hypoxia lowers CAT activity in carp muscles [47] and induces lipid oxidation in rat muscles [61]. In this study, feeding WEA reduced the levels of MDA and H_2_O_2_ in the carp’s gills while increasing the activity of CAT in their muscles and gills. Consistent findings from previous studies showed that dietary *Aloe vera* polysaccharides significantly reduced the quantity of MDA in the oral mucosa of oral ulceration [15]. GR converted oxidized GSH (GSSG) to normal GSH in order to preserve a high level of intracellular GSH [3]. This study found that dietary WEA enhanced GR activity in carp muscles. The effects of WEA on these parameters in fish muscles and gills are not well documented. These results suggest that WEA supplementation increases the antioxidant capacity in fish muscles and gills by suppressing the formation of ROS and lipid oxidation while enhancing enzymatic antioxidant activities. The results reported above suggest that dietary WEA supplementation improves fish hypoxia tolerance via boosting the antioxidant capacity of fish respiratory-related tissues and cells.

## 5. Conclusions

In conclusion, dietary WEA improves fish growth performance. The effects of WEA on growth performance are closely associated with increased antioxidant capacity in fish digestive organs. Dietary WEA also increases hypoxia tolerance in fish. Based on DT and OCR regression analysis, the optimal WEA addition levels for carp were found to be 1.217% and 1.220%, respectively. This work demonstrates that dietary WEA supplementation improves hypoxia tolerance by altering the metabolism of proteins and carbohydrates for energy production and boosting antioxidant defense in fish respiratory-related tissues and cells. According to this research, WEA may function as a natural regulator of hypoxia-induced stress in fish, offering a possible dietary approach to enhance fish health and tolerance in low-oxygen settings.

## Figures and Tables

**Table 1:** Composition of water extract of *Aloe vera* peel (WEA).

Ingredients	Content (%)
Dry matter	81.40 ± 0.43
Total sugar	39.34 ± 3.47
Reducing sugar	10.50 ± 1.01
Polysaccharide	28.84 ± 2.46

Values are means ± SD of 3 replicates.

**Table 2:** Composition and proximate analysis of experimental diets containing the water extract of *Aloe vera* peel (WEA).

Ingredients (%)	0.0%	0.3%	0.6%	0.9%	1.2%	1.5%	1.8%
Fish meal	22.00	22.00	22.00	22.00	22.00	22.00	22.00
Meat bone meal	5.00	5.00	5.00	5.00	5.00	5.00	5.00
Soybean meal	16.00	16.00	16.00	16.00	16.00	16.00	16.00
Corn gluten meal	7.00	7.00	7.00	7.00	7.00	7.00	7.00
Cottonseed meal	5.00	5.00	5.00	5.00	5.00	5.00	5.00
wheat middlings	14.00	14.00	14.00	14.00	14.00	14.00	14.00
Wheat flour	26.00	25.70	25.40	25.10	24.80	24.50	24.20
WEA	0.00	0.30	0.60	0.90	1.20	1.50	1.80
DL-methionine	0.60	0.60	0.60	0.60	0.60	0.60	0.60
Threonine	0.60	0.60	0.60	0.60	0.60	0.60	0.60
Fish oil	1.20	1.20	1.20	1.20	1.20	1.20	1.20
Corn oil	0.60	0.60	0.60	0.60	0.60	0.60	0.60
Vitamin mixture ^1^	1.00	1.00	1.00	1.00	1.00	1.00	1.00
Mineral mixture ^2^	1.00	1.00	1.00	1.00	1.00	1.00	1.00
Proximate analysis (%)							
Dry matter	94.28	94.53	94.21	94.49	94.35	94.51	94.25
Crude protein	34.89	34.68	34.94	34.77	34.71	35.02	34.83
Crude lipid	5.33	5.30	5.37	5.32	5.32	5.28	5.27
Ash	7.03	6.97	6.96	6.95	7.10	6.92	6.93

^1^ The vitamin mix per kilogram is shown below: Folic acid (96%), 0.52 g; cholecalciferol (500,000 IUg-1), 0.48 g; D-biotin (2%), 5.00 g; DL-α-tocopherol acetate (50%), 20.00 g; meso-inositol (99%), 52.33 g; thiamin nitrate (90%), 0.11 g; D-calcium pantothenate (90%), 2.73 g; niacin (99%), 2.82 g; riboflavine (80%), 0.63 g; cyanocobalamin (1%), 0.10 g; retinyl acetate (500,000 IUg^−1^), 0.80 g; ascorhyl acetate (93%), 7.16 g; and menadione (23%), 0.43 g. ^2^ The following displays the mineral combination per kilogram: CaCO_3_, 897.98 g; CuSO_4_·5H_2_O (25% Cu), 1.20 g; KI (4% I), 2.90 g; Na_2_SeO_3_·5H_2_O (1% Se), 2.50 g; ZnSO_4_·7H_2_O (23% Zn), 21.64 g; FeSO_4_·7H_2_O (20% Fe), 69.70 g; and MnSO_4_·H_2_O (32% Mn), 4.09 g.

**Table 3:** Impact of adding water extract of *Aloe vera* peel (WEA) to the diet on *Jian carp* growth performance over a 15-day period.

WEA (%)	IBW (g Fish^−1^)	FBW (g Fish^−1^)	FI (g Fish^−1^)	WGR (%)	FE (%)	PER
0.0	11.67 ± 0.29 ^a^	13.28 ± 0.22 ^a^	3.44 ± 0.19 ^a^	13.84 ± 1.31 ^a^	46.98 ± 4.36 ^a^	1.37 ± 0.13 ^a^
0.3	11.80 ± 0.07 ^a^	13.47 ± 0.04 ^ab^	3.44 ± 0.11 ^a^	14.17 ± 0.47 ^ab^	48.68 ± 2.18 ^a^	1.42 ± 0.06 ^a^
0.6	11.76 ± 0.03 ^a^	13.59 ± 0.18 ^abc^	3.55 ± 0.12 ^a^	15.64 ± 1.36 ^abc^	51.86 ± 6.37 ^ab^	1.52 ± 0.19 ^ab^
0.9	11.63 ± 0.28 ^a^	13.73 ± 0.13 ^bc^	3.47 ± 0.07 ^a^	18.08 ± 1.72 ^c^	60.50 ± 4.98 ^b^	1.77 ± 0.15 ^b^
1.2	11.92 ± 0.02 ^a^	13.85 ± 0.19 ^c^	3.51 ± 0.07 ^a^	16.25 ± 1.48 ^bc^	55.13 ± 4.87 ^ab^	1.61 ± 0.14 ^ab^
1.5	11.84 ± 0.03 ^a^	13.64 ± 0.18 ^bc^	3.48 ± 0.13 ^a^	15.18 ± 1.87 ^ab^	51.72 ± 6.15 ^ab^	1.51 ± 0.18 ^ab^
1.8	11.77 ± 0.02 ^a^	13.49 ± 0.16 ^ab^	3.44 ± 0.11 ^a^	14.67 ± 1.20 ^ab^	50.32 ± 4.98 ^a^	1.47 ± 0.15 ^a^

Values are means ± S.D. of 3 replicates, with 30 fish in each replicate. Values in the same column with different superscripts are significantly different (*p* < 0.05). IBW, initial body weight; FBW, final body weight; WGR, weight gain rate; FI, feed intake; FE, feed efficiency; PER, protein efficiency ratio.

**Table 4:** *Jian carp’s* hypoxia tolerance markers after 15 days of feed supplementation with water extract of *Aloe vera* peel (WEA).

WEA (%)	BW (g Fish^−1^)	WV (L Bottle^−1^)	IDO (mg L^−1^)	DT (h)	FDO (mg L^−1^)	OCR (mg g^−1^ h^−1^)
0.0	12.53 ± 1.15 ^a^	4.11 ± 0.38 ^a^	8.14 ± 0.00 ^a^	1.22 ± 0.04 ^a^	0.11 ± 0.02 ^a^	0.217 ± 0.005 ^b^
0.3	12.61 ± 0.63 ^a^	3.99 ± 0.17 ^a^	8.14 ± 0.00 ^a^	1.23 ± 0.06 ^a^	0.10 ± 0.01 ^a^	0.216 ± 0.015 ^b^
0.6	11.40 ± 1.30 ^a^	3.70 ± 0.49 ^a^	8.14 ± 0.00 ^a^	1.26 ± 0.05 ^a^	0.10 ± 0.01 ^a^	0.207 ± 0.004 ^b^
0.9	12.48 ± 0.92 ^a^	4.10 ± 0.37 ^a^	8.14 ± 0.00 ^a^	1.27 ± 0.08 ^a^	0.11 ± 0.02 ^a^	0.208 ± 0.007 ^b^
1.2	12.61 ± 1.73 ^a^	3.97 ± 0.23 ^a^	8.14 ± 0.00 ^a^	1.49 ± 0.06 ^b^	0.10 ± 0.01 ^a^	0.177 ± 0.012 ^a^
1.5	13.05 ± 1.18 ^a^	4.12 ± 0.03 ^a^	8.14 ± 0.00 ^a^	1.31 ± 0.05 ^ab^	0.11 ± 0.01 ^a^	0.201 ± 0.013 ^ab^
1.8	12.80 ± 1.04 ^a^	4.19 ± 0.21 ^a^	8.14 ± 0.00 ^a^	1.29 ± 0.09 ^a^	0.10 ± 0.01 ^a^	0.205 ± 0.021 ^ab^

The numbers show the means ± S.D. of three replicates, each with ten fish. There are significant variances (*p* < 0.05) between values with different superscripts in the same column. BW, body weight; OCR, oxygen consumption rate; WV, water volume; DT, durative time; FDO, final dissolved oxygen; IDO, initial dissolved oxygen.

**Table 5:** Effects of dietary water extract from *Aloe vera* peel (WEA) on the antioxidant indicators in the intestines and hepatopancreas of *Jian carp*.

WEA (%)	Intestines			Hepatopancreas	
	·OH (U mg^−1^ Protein)	SOD (U mg^−1^ Protein)	CAT (U mg^−1^ Protein)	H_2_O_2_ (mmol g^−1^ Protein)	GPx (U mg^−1^ Protein)
0.0	26.64 ± 8.55 ^c^	93.74 ± 12.28 ^a^	14.56 ± 0.59 ^a^	14.47 ± 1.66 ^c^	204.99 ± 22.75 ^a^
0.3	18.16 ± 1.48 ^ab^	111.84 ± 13.86 ^ab^	17.27 ± 1.06 ^b^	14.53 ± 0.82 ^c^	215.55 ± 8.42 ^ab^
0.6	12.35 ± 2.06 ^a^	120.77 ± 11.16 ^ab^	20.71 ± 1.47 ^c^	14.53 ± 2.90 ^c^	230.88 ± 29.43 ^ab^
0.9	11.22 ± 4.68 ^a^	123.91 ± 10.46 ^b^	19.81 ± 2.59 ^c^	10.86 ± 2.76 ^b^	231.24 ± 22.14 ^ab^
1.2	15.22 ± 2.94 ^ab^	125.06 ± 23.02 ^b^	17.79 ± 1.28 ^b^	10.070 ± 0.78 ^b^	245.56 ± 26.18 ^bc^
1.5	20.70 ± 3.76 ^bc^	116.10 ± 18.24 ^ab^	16.44 ± 1.24 ^ab^	7.45 ± 1.14 ^a^	291.31 ± 33.06 ^d^
1.8	21.30 ± 2.25 ^bc^	115.48 ± 16.13 ^ab^	15.39 ± 0.76 ^ab^	7.52 ± 1.06 ^a^	278.65 ± 37.86 ^cd^

The values represent the means ± S.D. of three replicates. Significant differences (*p* < 0.05) exist between values in the same column with different superscripts. ·OH, hydroxyl radical; SOD, superoxide dismutase; CAT, catalase; H_2_O_2_, hydrogen peroxide; GPx, glutathione peroxidase.

**Table 6:** Effects of dietary water extract from *Aloe vera* peel (WEA) on the hematological and hematochemical indicators in *Jian carp*.

WEA (%)	Hct (%)	RBC (10^12^ L^−1^)	HbC (g L^−1^)	MCV (fL Cell^−1^)	MCH (pg Cell^−1^)	MCHC (g L^−1^)	PAC (μmol L^−1^)
0.0	0.361 ± 0.012 ^a^	2.17 ± 0.10 ^a^	80.97 ± 4.65 ^a^	166.31 ± 6.25 ^a^	37.37 ± 2.74 ^a^	224.74 ± 14.71 ^a^	178.61 ± 5.44 ^c^
0.3	0.377 ± 0.020 ^ab^	2.15 ± 0.08 ^a^	84.87 ± 6.14 ^ab^	175.43 ± 11.96 ^ab^	39.53 ± 3.41 ^ab^	225.24 ± 9.06 ^a^	173.30 ± 9.62 ^c^
0.6	0.382 ± 0.015 ^ab^	2.16 ± 0.10 ^a^	83.98 ± 3.93 ^ab^	177.16 ± 12.38 ^ab^	38.95 ± 2.52 ^ab^	219.99 ± 6.66 ^a^	153.36 ± 5.69 ^b^
0.9	0.396 ± 0.008 ^b^	2.17 ± 0.17 ^a^	90.97 ± 6.83 ^b^	183.39 ± 13.07 ^b^	41.93 ± 0.60 ^b^	229.51 ± 15.33 ^a^	153.58 ± 9.73 ^b^
1.2	0.376 ± 0.015 ^ab^	2.18 ± 0.12 ^a^	87.37 ± 5.34 ^ab^	173.15 ± 10.33 ^ab^	40.26 ± 3.24 ^ab^	232.44 ± 10.72 ^a^	105.21 ± 6.43 ^a^
1.5	0.378 ± 0.019 ^ab^	2.18 ± 0.09 ^a^	84.20 ± 3.95 ^ab^	173.51 ± 12.36 ^ab^	38.71 ± 2.96 ^ab^	223.63 ± 18.65 ^a^	153.15 ± 6.56 ^b^
1.8	0.358 ± 0.014 ^a^	2.19 ± 0.12 ^a^	81.92 ± 4.73 ^a^	164.32 ± 9.93 ^a^	37.55 ± 2.39 ^a^	228.83 ± 13.69 ^a^	177.33 ± 10.59 ^c^

The values are the average ± SD of three replicates. Significant differences (*p* < 0.05) exist between values in the same column with different superscripts. PAC, plasma ammonia content; Hct, hematocrit; RBC, red blood cell count; HbC, hematocrit concentration; MCV, mean corpuscular volume; MCH, mean corpuscular hematocrit content; MCHC, mean corpuscular hematocrit concentration.

**Table 7:** Effects of dietary water extract from *Aloe vera* peel (WEA) on the antioxidant indicators in the red blood cells, gills, and muscles of *Jian carp*.

WEA (%)	Red Blood Cells			Gills			Muscles		
	O_2_^·−^ (U g^−1^ Protein)	H_2_O_2_ (mmol g^−1^ Protein)	GSH (mg g^−1^ Protein)	H_2_O_2_ (mmol g^−1^ Protein)	MDA (nmol mg^−1^ Protein)	CAT (U mg^−1^ Protein)	MDA (nmol mg^−1^ Protein)	CAT (U mg^−1^ Protein)	GR (U mg^−1^ Protein)
0.0	24.69 ± 1.17 ^cd^	45.39 ± 5.26 ^c^	6.06 ± 0.58 ^a^	60.10 ± 10.31 ^b^	14.80 ± 0.41 ^d^	4.76 ± 0.33 ^ab^	11.74 ± 1.02 ^b^	13.85 ± 0.94 ^a^	25.28 ± 5.70 ^ab^
0.3	24.32 ± 2.38 ^cd^	32.80 ± 6.44 ^ab^	7.49 ± 2.91 ^a^	49.89 ± 3.96 ^ab^	4.98 ± 0.28 ^a^	5.82 ± 0.25 ^b^	10.99 ± 0.96 ^ab^	14.59 ± 0.92 ^ab^	41.01 ± 5.81 ^c^
0.6	22.77 ± 0.54 ^bc^	33.26 ± 2.48 ^ab^	8.79 ± 1.81 ^a^	45.15 ± 10.32 ^a^	4.51 ± 0.90 ^a^	8.54 ± 1.00 ^c^	10.41 ± 1.08 ^ab^	14.77 ± 1.22 ^ab^	40.81 ± 6.21 ^c^
0.9	21.72 ± 1.13 ^b^	29.16 ± 1.27 ^a^	16.84 ± 2.89 ^c^	41.61 ± 3.44 ^a^	3.88 ± 0.35 ^a^	8.03 ± 1.12 ^c^	9.83 ± 1.31 ^a^	14.93 ± 0.83 ^ab^	46.63 ± 5.89 ^c^
1.2	19.20 ± 0.99 ^a^	34.24 ± 1.67 ^ab^	17.96 ± 3.26 ^c^	44.71 ± 3.55 ^a^	7.34 ± 1.63 ^b^	5.31 ± 0.90 ^b^	9.62 ± 0.32 ^a^	16.61 ± 0.71 ^c^	46.05 ± 8.46 ^c^
1.5	22.92 ± 1.22 ^bc^	35.53 ± 3.54 ^ab^	18.95 ± 1.91 ^c^	50.34 ± 3.57 ^ab^	11.25 ± 1.31 ^c^	4.60 ± 0.54 ^ab^	10.23 ± 0.70 ^ab^	16.28 ± 0.94 ^c^	31.90 ± 4.84 ^b^
1.8	26.12 ± 1.78 ^d^	37.49 ± 6.85 ^b^	12.60 ± 1.28 ^b^	50.19 ± 11.61 ^ab^	15.07 ± 2.34 ^d^	4.02 ± 0.24 ^a^	10.47 ± 0.96 ^ab^	15.67 ± 0.70 ^bc^	23.51 ± 3.81 ^a^

The values represent the means ± S.D. of three replicates. Significant differences (*p* < 0.05) exist between values in the same column with different superscripts. GSH, reduced glutathione; H_2_O_2_ hydrogen peroxide; O_2_^·−^, superoxide anion; CAT, catalase; MDA, malondialdehyde; GR, glutathione reductase.

## Data Availability

The original contributions presented in this study are included in the article. Further inquiries can be directed to the corresponding author.

## References

[1] Abdel-Tawwab M., Monier M.N., Hoseinifar S.H., Faggio C. (2019). Fish response to hypoxia stress: Growth, physiological, and immunological biomarkers. Fish Physiol. Biochem..

[2] Nikinmaa M. (2002). Oxygen-dependent cellular functions—Why fishes and their aquatic environment are a prime choice of study. Comp. Biochem. Physiol. Part A.

[3] Li H.T., Wu M., Wang J., Qin C.J., Long J., Zhou S.S., Yuan P., Jing X.Q. (2020). Protective role of *Angelica sinensis* extract on trichlorfon-induced oxidative damage and apoptosis in gills and erythrocytes of fish. Aquaculture.

[4] Remen M., Oppedal F., Torgersen T., Imsland A.K., Olsen R.E. (2012). Effects of cyclic environmental hypoxia on physiology and feed intake of post-smolt Atlantic salmon: Initial responses and acclimation. Aquaculture.

[5] Pollock M.S., Clarke L.M.J., Dubé M.G. (2007). The effects of hypoxia on fishes: From ecological relevance to physiological effects. Environ. Rev..

[6] Mustafa S.A., Al-Subiai S.N., Davies S.J., Jha A.N. (2011). Hypoxia-induced oxidative DNA damage links with higher level biological effects including specific growth rate in common carp, *Cyprinus carpio* L.. Ecotoxicology.

[7] Rosenfeld J., Lee R. (2022). Thresholds for reduction in fish growth and consumption due to hypoxia: Implications for water quality guidelines to protect aquatic life. Environ. Manag..

[8] Lushchak V.I., Lushchak L.P., Mota A.A., Hermes-Lima M. (2001). Oxidative stress and antioxidant defenses in goldfish *Carassius auratus* during anoxia and reoxygenation. Am. J. Physiol.-Regul. Integr. Comp. Physiol..

[9] Liu J., Ge Z., Jiang X., Zhang J., Sun J., Mao X. (2023). A comprehensive review of natural products with anti-hypoxic activity. Chin. J. Nat. Med..

[10] Chagas E.C., Val A.L. (2006). Ascorbic acid reduces the effects of hypoxia on the Amazon fish tambaqui. J. Fish Biol..

[11] Mandrioli R., Mercolini L., Ferranti A., Fanali S., Raggi M.A. (2011). Determination of aloe emodin in *Aloe vera* extracts and commercial formulations by HPLC with tandem UV absorption and fluorescence detection. Food Chem..

[12] Heidarieh M., Mirvaghefi A.R., Sepahi A., Sheikhzadeh N., Shahbazfar A.A., Akbari M. (2013). Effects of dietary *Aloe vera* on growth performance, skin and gastrointestine morphology in rainbow trout (*Oncorhynchus mykiss*). Turk. J. Fish. Aquat. Sci..

[13] Kwon K.H., Hong M.K., Hwang S.Y., Moon B.Y., Shin S., Baek J.H., Park Y.H. (2011). Antimicrobial and immunomodulatory effects of *Aloe vera* peel extract. J. Med. Plants Res..

[14] Kaithwas G., Singh P., Bhatia D. (2014). Evaluation of *in vitro* and *in vivo* antioxidant potential of polysaccharides from *Aloe vera* (*Aloe barbadensis* Miller) gel. Drug Chem. Toxicol..

[15] Yu Z., Jin C., Xin M., JianMin H. (2009). Effect of *Aloe vera* polysaccharides on immunity and antioxidant activities in oral ulcer animal models. Carbohydr. Polym..

[16] Mirghaed A.T., Fayaz S., Hoseini S.M. (2019). Effects of dietary 1,8-cineole supplementation on serum stress and antioxidant markers of common carp (*Cyprinus carpio*) acutely exposed to ambient ammonia. Aquaculture.

[17] Li S., Ji H., Zhang B., Zhou J., Yu H. (2017). Defatted black soldier fly (*Hermetia illucens*) larvae meal in diets for juvenile *Jian carp* (*Cyprinus carpio* var. Jian): Growth performance, antioxidant enzyme activities, digestive enzyme activities, intestine and hepatopancreas histological structure. Aquaculture.

[18] Zhou X.Q., Zhao C.R., Jiang J., Feng L., Liu Y. (2008). Dietary lysine requirement of juvenile *Jian carp* (*Cyprinus carpio* var. Jian). Aquac. Nutr..

[19] Li H.T., Lu L., Wu M., Xiong X.Q., Luo L., Ma Y.T., Liu Y. (2020). The effects of dietary extract of mulberry leaf on growth performance, hypoxia-reoxygenation stress and biochemical parameters in various organs of fish. Aquac. Rep..

[20] Li H.T., Yang D.D., Li Z.H., He M.Q., Li F.Y., Jiang J., Tang S.Y., Peng P.Y., Du W.H., Ma Y.T. (2019). Effects of *Angelica sinensis* extracts on lipid oxidation in fish feeds and growth performance of juvenile *Jian carp* (*Cyprinus carpio* var. Jian). Anim. Nutr..

[21] Ceylan B., Polat H., Küçük E., Ak O., Aydin İ. (2012). Optimum temperature and growth performance of hatchery reared Black Sea flounder (*Platichthys flesus luscus* Pallas, 1814). Turk. J. Vet. Anim. Sci..

[22] Ai Q.H., Mai K.S., Tan B.P., Xu W., Zhang W.B., Ma H.M., Liufu Z.G. (2006). Effects of dietary vitamin C on survival, growth, and immunity of large yellow croaker, *Pseudosciaena crocea*. Aquaculture.

[23] Cui Y.Y., Ma Q.Q., Limbu S.M., Du Z.Y., Zhang N.N., Li E.C., Chen L.Q. (2017). Effects of dietary protein to energy ratios on growth, body composition and digestive enzyme activities in Chinese mitten-handed crab, *Eriocheir sinensis*. Aquac. Res..

[24] Xu J., Chen G., Wu M., Yang Q.H., Li H.T. (2023). The extract of *Astragalus membranaceus* inhibits lipid oxidation in fish feed and enhances growth performance and antioxidant capacity in *Jian carp* (*Cyprinus carpio* var. Jian). Fishes.

[25] Long J., Yang P.Y., Liu Y.H., Liu X.R., Li H.T., Su X.Y., Zhang T., Xu J., Chen G.F., Jiang J. (2024). The extract of *Angelica sinensis* inhibits hypoxia–reoxygenation and copper-induced oxidative lesions and apoptosis in branchiae and red blood corpuscles of fish. Vet. Sci..

[26] Hong Y., Jiang W.D., Kuang S.Y., Hu K., Tang L., Liu Y., Jiang J., Zhang Y.A., Zhou X.Q., Feng L. (2015). Growth, digestive and absorptive capacity and antioxidant status in intestine and hepatopancreas of sub-adult grass carp *Ctenopharyngodonidella* fed graded levels of dietary threonine. J. Anim. Sci. Biotechnol..

[27] Jiang W.D., Feng L., Liu Y., Jiang J., Zhou X.Q. (2009). Myo-inositol prevents oxidative damage, inhibits oxygen radical generation and increases antioxidant enzyme activities of juvenile *Jian carp* (*Cyprinus carpio* var. Jian). Aquac. Res..

[28] Li H.T., Feng L., Jiang W.D., Liu Y., Jiang J., Li S.H., Zhou X.Q. (2013). Oxidative stress parameters and anti-apoptotic response to hydroxyl radicals in fish erythrocytes: Protective effects of glutamine, alanine, citrulline and proline. Aquat. Toxicol..

[29] Maqsood S., Benjakul S. (2010). Comparative studies of four different phenolic compounds on in vitro antioxidative activity and the preventive effect on lipid oxidation of fish oil emulsion and fish mince. Food Chem..

[30] Bradford M.M. (1976). A rapid and sensitive method for the quantitation of microgram quantities of protein utilizing the principle of protein-dye binding. Anal. Biochem..

[31] Darbkin D.L. (1946). Spectrophotometric studies. XIV the crystallographic and optical properties of the hemoglobin of man in comparison with these of other species. J. Biol. Chem..

[32] Jin M., Lu Y., Pan T., Zhu T., Yuan Y., Sun P., Zhou F., Ding X., Zhou Q. (2019). Effects of dietary n-3 LC-PUFA/n-6 C18 PUFA ratio on growth, feed utilization, fatty acid composition and lipid metabolism related gene expression in black seabream, *Acanthopagrus schlegelii*. Aquaculture.

[33] Choi J., Lee K.W., Han G.S., Byun S.-G., Lim H.J., Kim H.S. (2020). Dietary inclusion effect of krill meal and various fish meal sources on growth performance, feed utilization, and plasma chemistry of grower walleye pollock (*Gadus chalcogrammus*, Pallas 1811). Aquac. Rep..

[34] Syed R., Masood Z., Ul Hassan H., Khan W., Mushtaq S., Ali A., Gul Y., Jafari H., Habib A., Ishaq Ali Shah M. (2022). Growth performance, haematological assessment and chemical composition of Nile tilapia, *Oreochromis niloticus* (Linnaeus, 1758) fed different levels of *Aloe vera* extract as feed additives in a closed aquaculture system. Saudi J. Biol. Sci..

[35] Zhou X.Q., Zhao C.R., Lin Y. (2007). Compare the effect of diet supplementation with uncoated or coated lysine on juvenile *Jian Carp* (*Cyprinus carpio* var. Jian). Aquac. Nutr..

[36] Liu Y., Feng L., Jiang J., Liu Y., Zhou X.Q. (2009). Effects of dietary protein levels on the growth performance, digestive capacity and amino acid metabolism of juvenile *Jian carp* (*Cyprinus carpio* var. Jian). Aquac. Res..

[37] Li H.T., Liu H.J., Wu S.Y., Ai C.Y., Yang Q.H., Jia J.T., Xu X., Wu M., Jiang J. (2024). Effects of ferulic acid on respiratory metabolism, oxidative lesions, and apoptotic parameters in gills and red blood cells of carp (*Cyprinus carpio* var. Jian) response to copper. Antioxidants.

[38] Martínez-Álvarez R.M., Morales A.E., Sanz A. (2005). Antioxidant defenses in fish: Biotic and abiotic factors. Rev. Fish Biol. Fish..

[39] Feng L., Zhao S., Chen G.F., Jiang W.D., Liu Y., Jiang J., Hu K., Li S.H., Zhou X.Q. (2014). Antioxidant status of serum, muscle, intestine and hepatopancreas for fish fed graded levels of biotin. Fish Physiol. Biochem..

[40] Ni M., Liu M., Lou J., Mi G., Yuan J., Gu Z. (2021). Stocking density alters growth performance, serum biochemistry, digestive enzymes, immune response, and muscle quality of largemouth bass (*Micropterus salmoides*) in in-pond raceway system. Fish Physiol. Biochem..

[41] Foos A., Evensen T.H., Øiestad V. (2002). Effects of hypoxia and hyperoxia on growth and food conversion efficiency in the spotted wolffish Anarhichas minor (Olafsen). Aquac. Res..

[42] Braun N., de Lima R.L., Moraes B., Loro V.L., Baldisserotto B. (2006). Survival, growth and biochemical parameters of silver catfish, *Rhamdia quelen* (Quoy & Gaimard, 1824), juveniles exposed to different dissolved oxygen levels. Aquac. Res..

[43] Lushchak V.I., Bagnyukova T.V. (2007). Hypoxia induces oxidative stress in tissues of a goby, the rotan *Perccottus glenii*. Comp. Biochem. Physiol. Part B.

[44] Storey K.B., Storey J.M. (2007). Metabolic rate depression in animals: Transcriptional and translational controls. Biol. Rev..

[45] Witeska M., Kondera E., Lipionoga J., Jastrzębska A. (2010). Changes in oxygen consumption rate and red blood parameters in common carp *Cyprinus carpio* L. after acute copper and cadmium exposures. Fresenius Environ. Bull..

[46] Zhang W., Cao Z.D., Fu S.J. (2012). The effects of dissolved oxygen levels on the metabolic interaction between digestion and locomotion in Cyprinid fishes with different locomotive and digestive performances. J. Comp. Physiol. B.

[47] Lushchak V.I., Bagnyukova T.V., Lushchak O.V., Storey J.M., Storey K.B. (2005). Hypoxia and recovery perturb free radical processes and antioxidant potential in common carp (*Cyprinus carpio*) tissues. Int. J. Biochem. Cell Biol..

[48] Eliason E.J., Higgs D.A., Farrell A.P. (2007). Effect of isoenergetic diets with different protein and lipid content on the growth performance and heat increment of rainbow trout. Aquaculture.

[49] Li H.T., Wu M., Jiang J., Sun X.M., Chen L.J., Feng M., Yuan D.Y., Wen Z.Y., Qin C.J. (2019). The extracts of *Angelica sinensis* restore the digestive and absorptive capacity through improving antioxidant status in digestive organs of fish treated with trichlorfon. Aquac. Res..

[50] Metón I., Mediavilla D., Caseras A., Cantó E., Fernández F., Baanante I.V. (1999). Effect of diet composition and ration size on key enzyme activities of glycolysis–gluconeogenesis, the pentose phosphate pathway and amino acid metabolism in liver of gilthead sea bream (*Sparus aurata*). Br. J. Nutr..

[51] Sinha A.K., Giblen T., AbdElgawad H., De Rop M., Asard H., Blust R., De Boeck G. (2013). Regulation of amino acid metabolism as a defensive strategy in the brain of three freshwater teleosts in response to high environmental ammonia exposure. Aquat. Toxicol..

[52] Saha S.K., Pathak N.N. (2021). Fundamentals of Animal Nutrition.

[53] Shiau S.Y., Peng C.Y. (1993). Protein-sparing effect by carbohydrates in diets for tilapia, *Oreochromis niloticus* × *O. Aureus*. Aquaculture.

[54] Cimen M.Y. (2008). Free radical metabolism in human erythrocytes. Clin. Chim. Acta.

[55] Speckner W., Schindler J.F., Albers C. (1989). Age-dependent changes in volume and haemoglobin content of erythrocytes in the carp (*Cyprinus carpio* L.). J. Exp. Biol..

[56] Johnson R.M., Goyette G., Ravindranath Y., Ho Y.S. (2005). Hemoglobin autoxidation and regulation of endogenous H_2_O_2_ levels in erythrocytes. Free Radic. Biol. Med..

[57] Winston G.W., Giulioz R.T.D. (1991). Prooxidant and antioxidant mechanisms in aquatic organisms. Aquat. Toxicol..

[58] Sinha A.K., Zinta G., AbdElgawad H., Asard H., Blust R., De Boeck G. (2015). High environmental ammonia elicits differential oxidative stress and antioxidant responses in five different organs of a model estuarine teleost (*Dicentrarchus labrax*). Comp. Biochem. Physiol. Part C Toxicol. Pharmacol..

[59] Xiong X.Y., Huang G.Q., Peng Y.H., Liu X.J. (2016). Effect of hypoxia on growth performance, energy metabolism and oxidative stress of *Mugil cephalus*. J. Fish. China.

[60] Shadwick R.E., Syme D.A. (2008). Thunniform swimming: Muscle dynamics and mechanical power production of aerobic fibres in yellowfin tuna (*Thunnus albacares*). J. Exp. Biol..

[61] Gaurava, Praveenvats, Shyam R., Suri S., Kumria M., Sridharan K., Sharmaa P., Singh S.N. (2001). Effect of pomegranate (*Punica granatum* L.) juice on changes in tissue glutathione levels of rats exposed to high altitude hypoxia. Anc. Sci. Life.

